# Migrated tubal sterilisation clip presenting as a subcutaneous gluteal foreign body 24 years later: a case report and literature review

**DOI:** 10.1186/s40792-024-01937-3

**Published:** 2024-06-12

**Authors:** Adil S. Lakha, Andrew Ang, Sarmad Mohammed Salih, Christopher Lewis

**Affiliations:** Surgical Emergency Unit, John Radcliffe Hospital, Oxford University Hospitals NHS Foundation Trust, Headington, Oxford, OX3 9DU UK

**Keywords:** Sterilisation, Filshie, Clip migration, Tubal ligation, Subcutaneous foreign body, Pelvic floor, Tubal clip migration

## Abstract

**Background:**

The incidence of sterilisation clip migration is reportedly 25%. However, less than 1% of those who experience clip migration will present with pain, an abscess, or spontaneous extrusion. Here we present a rare case of sterilisation clip migration through the entire pelvic floor.

**Case presentation:**

A 66-year-old female was referred from community to the Surgical Emergency Unit with a possible metallic foreign body under the skin following an attempted routine gluteal cyst excision. The patient first noticed a lump under the skin 2 years ago which gradually became more apparent and tender over the previous 2 months. The patient denied recent trauma, had no co-morbidities and had a sterilisation procedure 24 years prior. Examination revealed a non-mobile solid structure just beneath the skin 5 cm laterally from the anal verge. Inflammatory markers were normal and an ultrasound confirmed a 15 × 7 mm foreign body in the subcutaneous tissues. The foreign body was excised easily under local anaesthesia, revealing a closed Filshie sterilisation clip. The wound was closed primarily, and recovery was uncomplicated.

**Conclusions:**

This was a case of sterilisation clip migration to the subcutaneous gluteal region. A literature review revealed 34 case reports of sterilisation clip migration, mostly to the bladder. Patients with a previous sterilisation procedure and suspected subcutaneous foreign body without trauma should elicit a high index of suspicion for migrated sterilisation clips. These clips can migrate through multiple layers of muscle and fascia, including the pelvic floor.

## Background

The incidence of tubal clip migration is reportedly 25%. However, less than 1% of those who experience clip migration will present with symptoms such as pain, an abscess, or spontaneous extrusion [1]. As we report in this article, clip migration is unpredictable, rare, and the exact mechanisms are not well understood. Here, we present a rare case of sterilisation clip migration through the entire pelvic floor, as well as a literature review of reported sterilisation clip migration.

## Case

**Fig. 1:**
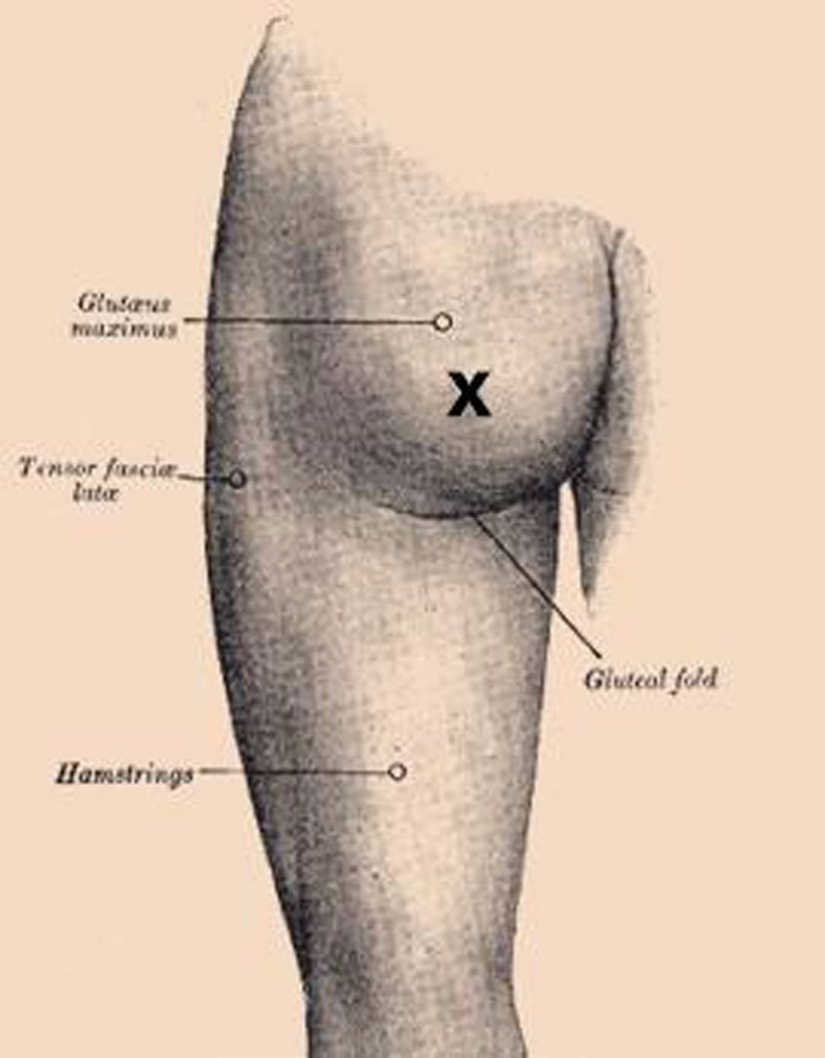
Illustration of location of foreign body palpated during examination, marked with a cross, on an image adapted from https://theodora.com/anatomy/surface_anatomy_of_the_lower_extremity.html

**Fig. 2:**
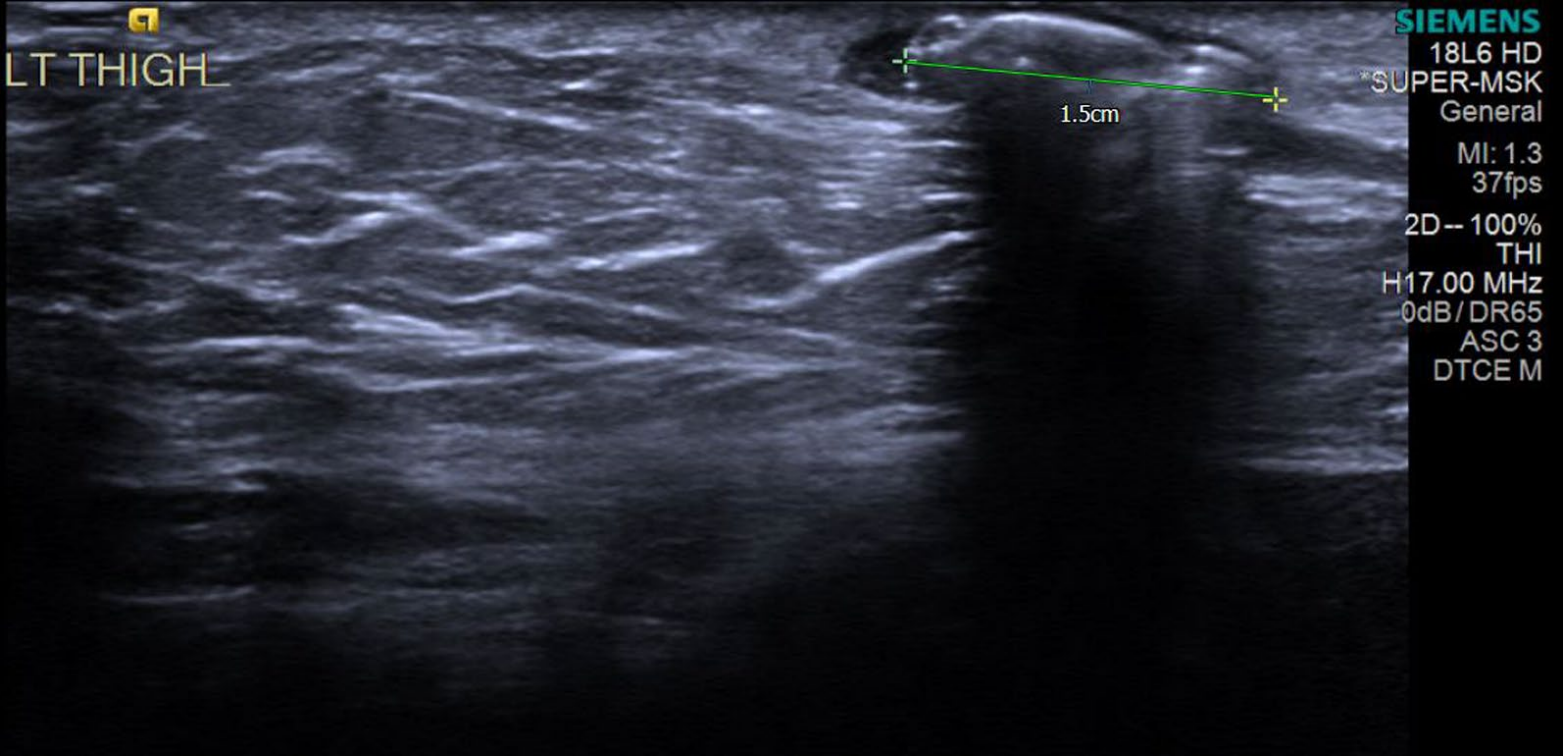
Admission ultrasound image showing foreign body in the subcutaneous tissue of the posteromedial left thigh

A 66-year-old female was referred from the community to the Surgical Emergency Unit with a possible metallic foreign body under the skin following an attempted routine gluteal cyst excision. The patient first noticed a lump under the skin 2 years ago which gradually became more apparent and tender over the past 2 months. The patient denied recent trauma or procedures in the gluteal region and inflammatory markers were normal. Examination revealed a non-mobile solid pin-like structure just beneath the skin 5 cm laterally from the anal verge (Fig. 1). An ultrasound confirmed a 15 × 7 mm foreign body in the subcutaneous tissue of the posteromedial aspect of the upper thigh (Fig. 2). The foreign body was excised easily under local anaesthesia, revealing a closed Filshie sterilisation clip. The wound was closed primarily, and recovery was uncomplicated. Apart from a laparoscopic sterilisation procedure 24 years prior, the patient was fit and well, with a normal body mass index (BMI).

## Discussion

The overall number of sterilisation procedures in NHS hospitals is falling. The latest NHS Digital data suggest a 26% decrease in annual sterilisation procedures over a 10-year period. In the 2018/2019 year, there were 12,918 procedures performed, down from 17,562 in 2008/2009 [2]. However, there is a paucity of population-wide data on the specific type of female sterilisation method used. There have been advances in sterilisation methodology over the last two decades, namely the introduction of transcervical hysteroscopic devices. However, laparoscopic and laparotomic approaches are still utilised particularly in developing countries to achieve the common goal of uterine tubal occlusion [3]. As with all surgical procedures, this operation carries risks. Specific risks include a failed procedure, migration of the clip to other structures, and damage to surrounding anatomy. The latest NICE guidance corroborates a 1 in 200 failure rate of this procedure in achieving sterilisation [4], as reported in a 2001 publication by Mr Marcus Filshie, inventor of the Filshie clip [5]. With regard to clip migration, it appears that slow peritonealisation of the clip(s) results in a higher likelihood of migration. Faster peritonealisation of the clips renders them more likely to remain in situ [5]. Furthermore, while allergic reactions have been reported to cause Hulka clip sterile abscess formation [6], the titanium-silicone basis of Filshie clips makes this less likely, since these materials are commonly implanted elsewhere in the body and are not generally associated with sterile abscess formation [7]. The exact mechanism of migration through the pelvic floor however remains poorly understood. Given the rarity of this pathology, there is limited information on the pathophysiology of this migratory behaviour. Principles of acute and chronic inflammation in Crohn’s disease may help to explain how an abscess from the fallopian tubes could form a fistulous tract through the pelvic floor through neutrophil, macrophage, and fibroblast action, however this is unproven and requires further investigation [8].

**Fig. 3:**
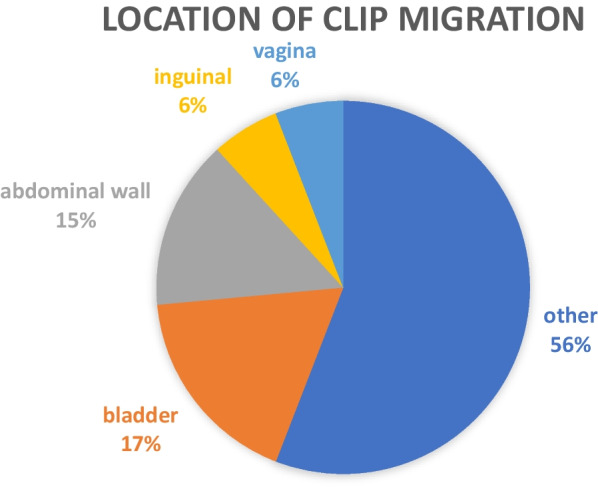
Proportion of reports of migration by anatomical location

**Fig. 4:**
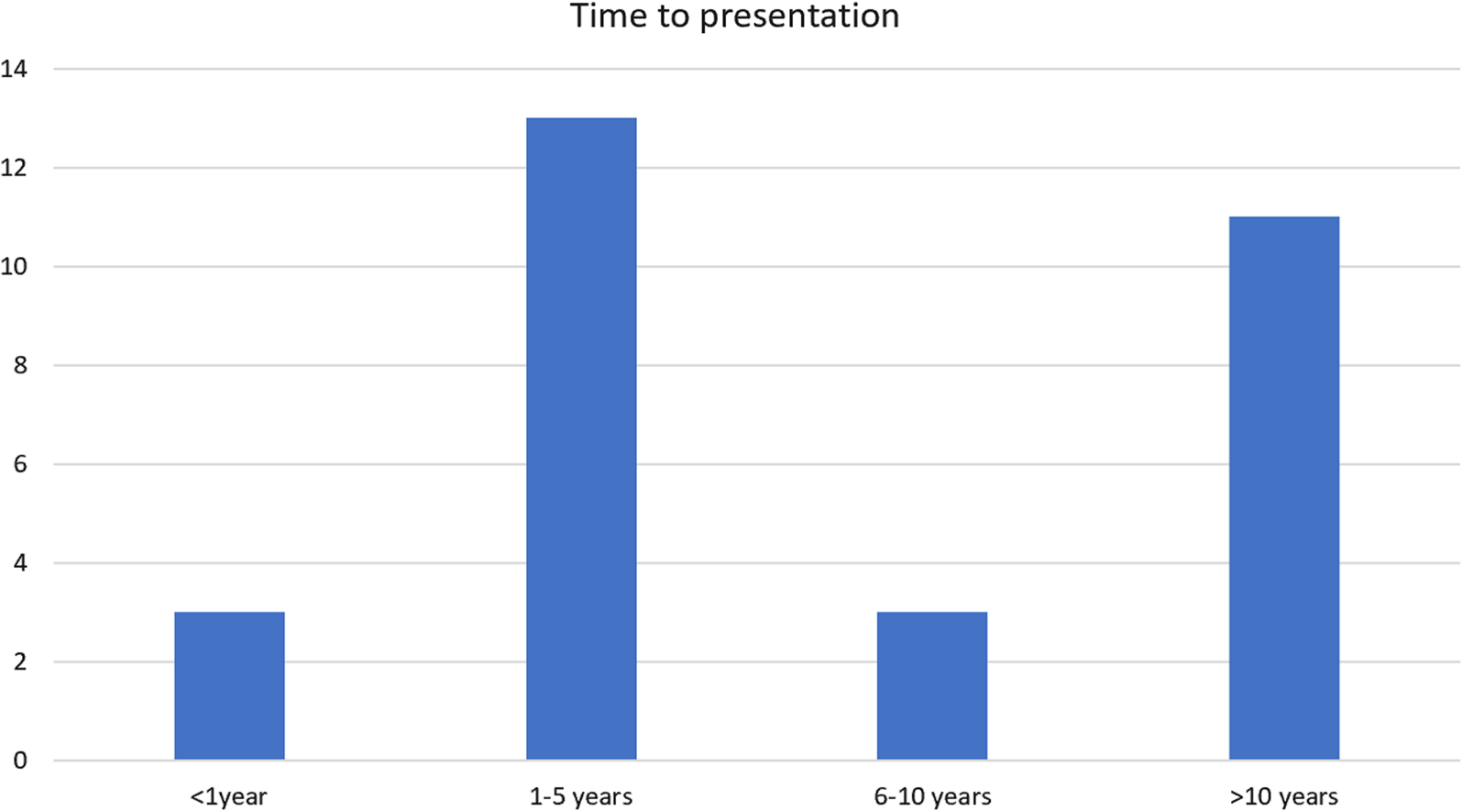
Variation in time to acutely presenting with clip migration symptoms following sterilisation procedure

We performed a literature review (Table 1) to determine whether this was a unique migration location, as well as to ascertain the incidence of sterilisation clip migration. In June 2023, we searched PubMed from June 1950 to June 2023 with the search terms [(sterilisation) OR (tubal ligation)] AND (clip) AND (migration) to identify other cases of sterilisation clip migration. We also searched the references of each of these papers to further widen our capture. This revealed 34 case reports of sterilisation clip migration, mostly to the bladder and anterior abdominal wall (Fig. 3). Despite an extensive literature search, no similar cases to this were identified. Locations such as the bladder, pelvic peritoneum, inguinal region, femoral region, anterior abdominal wall, spontaneous expulsion through the vagina, and even the liver have all been reported, however none to the gluteal area through the pelvic floor. Interestingly, this complication of tubal sterilisation does not appear to be either an early (< 5 years) or late (> 10 years) event. Figure 4 illustrates how there is a range of when these patients have presented in the acute setting for treatment. However, when exactly the migration occurs is not yet known, as a proportion of these cases may well show migration prior to acute illness presentation; for example, those with asymptomatic migration presenting later with acute sepsis. Furthermore, not all cases of clip migration will have been published in the literature, and there may be further cases not captured by our search strategy, therefore the true prevalence of these instances may be underestimated. Clips placed elsewhere in the body may also migrate. Surgical clip migration after laparoscopic cholecystectomy is another documented occurrence of a different type of clip migration [9]. A case report described a patient who developed cholangitis due to clip migration 10 years after initial laparoscopic cholecystectomy. CT imaging revealed hyperdense material in the dilated common bile duct, retrieved by endoscopic retrograde cholangiopancreatography via balloon trawl.

**Table 1:** Literature review of sterilisation clip migration

Study	Age	Diagnosis	Clip type	Migration location	Time interval	Outcome
Frigenza et al. 2012 [10]	52	MRI uterus	Filshie	Posterior to psoas	16 years	Recurrent I + D (× 5) for abscesses, eventually removed
Poo et al. 2020 [11]	52	CT abdomen	Filshie	Bladder wall	12 years	Diagnostic laparoscopy, converted to an exploratory laparotomy due to dense omental adhesions. A Filshie clip was found within the bladder wall abscess and removed
Verma et al. 2007 [12]	53	-	Filshie	Right-sided extraperitoneal abscess	13 years	Exploratory laparotomy demonstrating right-sided extraperitoneal abscess containing 400 mL pus and a Filshie clip
Daucher et al. 2006 [13]	25	Diagnostic laparoscopy	Filshie	Peritoneal surface of the abdominal wall + bladder	2 years	Removed at diagnostic laparoscopy
Kolias 2010 [14]	56	–	Filshie	Chronic groin sinus	21 years	Exploration and sinus excision
Dua et al. 2007 [15]	45	EUA MRI	Filshie	Ischiorectal	4 years	Debridement of cavity, corrugated drain in situ, packing Subsequent MRI—two fistulous tracts
Mumme et al. 2015 [16]	56	CT	Filshie	Right femoral	2 years	Right fem hernia repair and clip removal
Hasan et al. 2005 [17]	38	EUA	Filshie	Low fistula in ano	12 years	Low fistula in ano was opened, and a Filshie clip was found lying across the abscess cavity
Konate 2002 [18]	44	–	Filshie	Intraperitoneal	5 years	Clip ablation
Berendst 1994	35	–	Filshie	Right inguinal	5 years	Surgical exploration of right inguinal abscess
Klumper et al. 1991 [19]	–	Diagnostic laparoscopy	Filshie	-	–	–
Tan 2004 [20]	–	CT	Filshie	Subcutaneous tissue at anterior abdominal wall with surrounding granuloma and abscess formation	2 years	Laparotomy
Kalu 2006 [21]	35	Diagnostic laparoscopy	Filshie	Peritoneal defect in the broad ligament lateral to the left uterosacral ligament. Clip deeply embedded and adherent to the pelvic peritoneum	3 years	Diagnostic laparoscopy, where the clip was found with jaws closed. It was removed from the pelvis using bipolar diathermy
Kesby et al. 1997 [22]	49	Presented with macroscopic haematuria, passed the clip whilst urinating	Filshie	Mobile left fallopian tube transected approximately 1–1.5 cm from the left uterine cornu, with a Filshie clip loosely attached to peritoneum at the site of tubal separation	7 years	Diagnostic laparoscopy. The clip was removed through the 5 mm side-port
Husemeyer 1999 [23]	–	-	Filshie	Anterior abdominal wall	5 years	Spontaneous expulsion of a Filshie clip
Sharma et al. 2020 [1]	49	AXR, CT	Filshie	Underneath the left lobe of the liver embedded in the gastrohepatic omentum	20 years	Laparoscopy for removal of the clip
Fahey 2007 [24]	–	–	Hulka	Extrusion with associated tuboperitoneal fistula	–	Following two surgical procedures to excise fistulous tracts, the patient spontaneously expelled three Hulka tubal ligation clips from the vagina
Siew 1991 [25]	35	Hysterosalpingogram showed bilateral blockage of the tubes and one clip on the left side	Hulka	–	4 years	Clamped Hulka clip that had been passed through the vagina
Saha et al. 2006 [26]	36	Ultrasound abdomen revealed a 6 × 10 cm thick-walled, septated, cystic mass extending from above the fundus of the uterus along the anterior abdominal wall	Filshie	Bladder/omentum	6 years	Exploratory laparotomy. Filshie clip was found within a pus-filled cavity of about 8 cm diameter with thick walls. The mass was also adherent to the bladder dome and the omentum
Goodden 1993 [24]	21	X-ray—two missing on left fallopian tube	Hulka	Vagina	17 months	Spontaneous, asymptomatic passage of two Hulka clips into the vagina
Pandit 2005 [27]	37	–	Filshie	Transperitoneal migration to rectum	8 weeks	Spontaneous passage per rectum
Loddo et al. 2008 [28]	44	AXR	Filshie	Pouch of Douglas	6 years	–
Palanivelu et al. 2007 [29]	37	EUA, cystoscopy, diagnostic laparoscopy	Filshie	Urethra	18 months	–
Kale et al. 2008 [30]	32	–	Filshie	Vagina	5 years	Spontaneous vaginal expulsion
Krishnamoorthy et al. 2004 [31]	–	–	Filshie	Anterior abdominal wall	–	Spontaneous extrusion
Miliauskas 2003 [32]	–	–	Filshie	Bladder	–	–
Gad et al. 2010 [33]	40	Diagnostic laparoscopy	Filshie	Bladder	10 years	Removed during laparoscopy without complication
Connolly et al. 2004 [34]	–	–	Filshie	Bladder	10 years	Spontaneous extrusion through the urethra
Lok et al. 2003 [35]	–	–	Filshie	Sub-umbilical anterior abdominal wall	5 years	Spontaneous extrusion from abscess and subsequent removal
Buczaki et al. 2007 [33]	–	–	Filshie	Pararectal tissues	15 years	Surgical removal
Garner et al. 1998 [36]	–	–	Filshie	Femoral hernia	5 years	Surgical removal of 3 clips in femoral hernia sac
Khalil et al. 2006 [37]	–	–	Filshie	Groin with inguinal sinus formation	20 years	Spontaneous expulsion per inguinal sinus
Verma et al. 2007 [12]	–	–	Filshie	Right groin with extraperitoneal abscess formation	13 years	Surgical removal
Denton et al. 1990 [38]	35	–	Filshie	Appendiceal lumen	2 years	Surgical removal

Patients with a previous sterilisation procedure and suspected subcutaneous foreign body without trauma should elicit a high index of suspicion for migrated sterilisation clips. We show that these clips can migrate through multiple layers of muscle and fascia, including the pelvic floor. Clinicians should be aware of the possibility of clip migration especially when patients present with recurrent abscesses of unknown cause, and thorough history taking may help to guide the working diagnosis. The role of plain film X-radiography is yet to be elucidated, however this may be a simple screening investigation if a radiopaque foreign body is thought to be the underlying cause. Ultimately, the majority of the cases of sterilisation clips were diagnosed on surgical exploration, rather than prior imaging. However, in cases of recurrent abscesses, pre-operative imaging may help reduce the incidence of recurrent abscess formation especially in cases where the sterilisation clip is not excised during the index operation.

## Conclusions

Patients with a previous sterilisation procedure and suspected subcutaneous foreign body without trauma should elicit a high index of suspicion for migrated sterilisation clips. These clips can migrate through multiple layers of muscle and fascia, including the pelvic floor.

## Data Availability

All data available are presented.

## Abbreviations

BMI: Body mass index
NHS: National Health Service
MRI: Magnetic resonance imaging
CT: Computerised tomography
I + D: Incision and drainage
EUA: Examination under anaesthetic
AXR: Abdominal X-ray
